# Stem Cell-Based Approaches for the Treatment of Diabetes

**DOI:** 10.4061/2011/424986

**Published:** 2011-05-18

**Authors:** Catriona Kelly, Cara C. S. Flatt, Neville H. McClenaghan

**Affiliations:** ^1^SAAD Centre for Pharmacy & Diabetes, Biomedical Sciences Research Institute, School of Biomedical Sciences, University of Ulster, Coleraine BT52 1SA, UK; ^2^Institute for Science & Technology in Medicine, Keele University, Keele ST5 5BG, UK

## Abstract

The incidence of diabetes and the associated debilitating complications are increasing at an alarming rate worldwide. Current therapies for type 1 diabetes focus primarily on administration of exogenous insulin to help restore glucose homeostasis. However, such treatment rarely prevents the long-term complications of this serious metabolic disorder, including neuropathy, nephropathy, retinopathy, and cardiovascular disease. Whole pancreas or islet transplantations have enjoyed limited success in some individuals, but these approaches are hampered by the shortage of suitable donors and the burden of lifelong immunosuppression. Here, we review current approaches to differentiate nonislet cell types towards an islet-cell phenotype which may be used for larger-scale cell replacement strategies. In particular, the differentiation protocols used to direct embryonic stem cells, progenitor cells of both endocrine and nonendocrine origin, and induced pluripotent stem cells towards an islet-cell phenotype are discussed.

## 1. The Need for Islet Cell Replacement Strategies

The World Health Organisation (WHO) estimates that 220 million people suffer from diabetes worldwide, while approximately 3.4 million individuals died as a result of hyperglycaemic complications in 2004. Administration of exogenous insulin is the fundamental means of treating hyperglycaemia in type 1 diabetes, but it does not restore the physiological regulation of blood glucose. Additionally, patients with poorly controlled type 2 diabetes are increasingly being prescribed insulin therapy, with studies suggesting that intensive insulin therapy even in newly diagnosed type 2 diabetes can improve beta-cell survival and function compared with oral hypoglycaemic agents [1]. However, tight glycaemic control, with its inherent risk of hypoglycaemia, is required to prevent many of the long-term complications of diabetes including cardiovascular disorders, nephropathies, and diabetic retinopathy. WHO figures show that 50% of people with diabetes die of cardiovascular disease, while kidney failure accounts for 10–20% of deaths. Given these shortcomings, recent research has been directed towards establishing cellular-based therapies that avoid the need for exogenous insulin delivery by conventional injection or more modern pump technology (see the study by Cohen and Shaw [2]). 

Arguably one of the most attractive of these strategies involves replacement of insulin-producing islet-cells by transplantation therapy [3, 4]. The first successful transplantation of isolated pancreatic islets was conducted in rodents by Ballinger and Lacy in 1972 [5]. Although this study offered hope that a cure for diabetes was possible, four decades later, islet transplantation in humans is not commonplace. The lack of fresh viable donor material coupled with problems of immunocompatability and life-long immunosuppression to prevent graft rejection has made the widespread application of both techniques almost impossible [3, 4, 6]. 

Stem cells are found in multicellular organisms and have the potential to differentiate into a variety of different cell types. Stem cells are largely divided according to their potency or ability to differentiate. Totipotent stem cells may generate any somatic or germline cell, while pluripotent stem cells may give rise to cells originating from any of the three germ layers: endoderm, mesoderm, or ectoderm. The current paper examines advances in the field of stem cell therapy for the treatment of diabetes and outlines the varied approaches that have been used to create insulin-producing cells. In particular, the exploitation of developmental biology pathways, which are briefly outlined in the following, to direct embryonic stem cells (ESCs) towards an insulin-producing phenotype is examined. Alternative approaches including the use of pancreatic adult stem cells, islet progenitor cells of both endocrine and nonendocrine origin, and induced pluripotent stem cells are also considered.

## 2. Development of the Endocrine Pancreas

**Figure 1:**
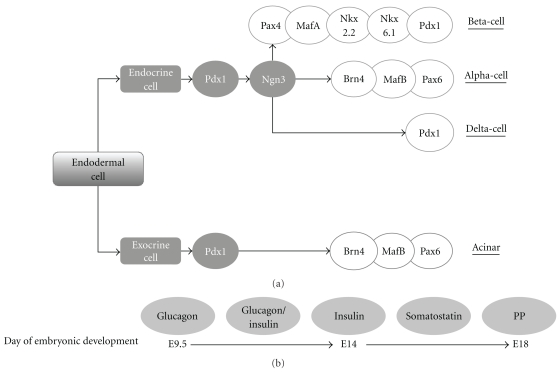
Regulation of pancreatic development. (a) Pancreatic cells (both endocrine and exocrine) originate from the same Pdx-1 expressing endodermal cells. The transcription factor Ngn3 is required for differentiation into an endocrine phenotype. Further development into insulin-, glucagon-, or somatostatin-secreting cells is tightly regulated by a range of transcription factors as indicated in the figure. Pax, NKX, Pdx-1, and Brn4 are homeodomain proteins which are generally involved in morphogenesis, while MafA and MafB are members of the large Maf protein family which regulates pancreatic development. (b) Timescale showing emergence of islet hormone-producing cells in the rodent embryo.

The pancreas is formed during embryogenesis from fusion of the dorsal and ventral primordia and has both exocrine and endocrine functions [7]. The transcriptional regulation of pancreas differentiation is shown in Figure 1. The adult human pancreas is comprised of approximately 1 million islets of Langerhans that form the endocrine portion of the gland, constituting 2-3% of the total pancreatic mass [8]. Acinar and ductal tissues largely comprise the exocrine pancreas. Islets are anatomically complex microorgans comprised of heterogenous cell types that secrete insulin from the beta-cell, glucagon from the alpha-cell, somatostatin from the delta-cell, and pancreatic polypeptide (PP) from PP cells [8].

During differentiation of the endocrine tissue, progenitor cells coexpress various endocrine hormones prior to final maturation into cells expressing a single hormone [7]. In rodent models, the first endocrine cells detected are glucagon-secreting cells which are evident from approximately embryonic day 9.5 [9, 10]. This is followed by the presence of insulin-producing cells which coexpress glucagon, while fully differentiated insulin-releasing beta-cells and glucagon-secreting alpha-cells are observed from day 14. Somatostatin-secreting delta-cells develop soon afterwards, while PP-releasing cells are observed shortly before the end of gestation at approximately day 18 when clustering of cells to form islets is also observed [9, 10]. 

All endocrine cells originate from pancreatic and duodenal homeobox 1 (Pdx-1) expressing progenitor cells (see Figure 1). During pancreatic development, Pdx-1 is expressed in both endocrine and exocrine progenitors, but, in the developed pancreas, Pdx-1 is generally observed in the beta-cells and delta-cells [11]. The development of endocrine cells is regulated by the basic helix-loop-helix (bHLH) transcription factor Neurogenin 3 (Ngn3), with the inhibition of Ngn3 in the pancreas at embryonic day 11.5 resulting in a significant reduction in endocrine differentiation [12]. The further development of specific islet hormone-expressing cells is regulated by an additional range of transcription factors [13]. In particular, beta-cell differentiation is largely regulated by NK2 homeobox 2 (NKX2.2) [14]. For a more complete review of pancreatic development, please see the study by Kordowich et al. [15].

## 3. Endocrine Progenitor Cells and Beta-Cell Replication

Ngn3 is essential for the development of all endocrine cells from Pdx-1-positive progenitors [13]. Ngn3 null mice lack pancreatic islets and die of hyperglycaemia shortly after birth [13]. Although generally associated with the developing pancreas, it has been shown that adult islet cells express low levels of Ngn3 and that this may play a role in regulating beta-cell replication [16].

Beta-cells are capable of responding to changing physiological requirements including pregnancy and obesity which are associated with an increase in beta-cell mass and insulin secretion [8, 17]. Animal studies have shown that beta-cells self-duplicate (see review by Gonez and Knight [18]), although the mechanisms behind this process are poorly understood. Various compounds have been shown to enhance beta-cell proliferation and mass. Most notably, treatment with gastrin and epidermal growth factor (EGF) has been shown to evoke significant improvements in the number of insulin-producing cells present [19]. However, the effects of such treatment are transient and short-lasting. Therefore, the therapeutic use of beta-cells to create other beta-cells is complicated and unlikely to be applicable on a long-term basis *in vivo*. 

The regeneration of beta-cells by existing beta-cells takes place in association with pancreatic ductal cells [20]. However, debate exists as to whether ductal cells are true progenitors for the development of pancreatic beta-cells. A recent study labeled pancreatic ductal cells following duct ligation in mice and concluded that these labeled cells differentiated into beta-cells after 4 weeks [21]. Similar studies have confirmed this result [22]. However, as a note of caution, other work contradicts these findings and suggests that ductal cells contribute to beta-cell neogenesis before, but not after, birth [23, 24]. The presence of true endocrine progenitors in the adult pancreas is, therefore, highly debatable, and many argue that the relative lack of constitutive Ngn3 expression in adult endocrine cells would suggest that beta-cell neogenesis originates from existing beta-cells. However, work by Finegood and colleagues showed that the rat pancreas was able to regenerate even after extreme conditions of 90% pancreatectomy and pretreatment with the beta-cell toxin streptozotocin [25]. This would suggest that beta-cells are not the only source of beta-cell neogenesis in the adult islet. Moreover, treatment of pancreatic ductal cells with Glucagon-like peptide 1 (GLP-1) enhances beta-cell proliferation and reduces apoptosis, *in vivo* and *in vitro* [26]. Activation of the GLP-1 receptor is thought to improve islet neogenesis by upregulating Pdx-1 expression in ductal progenitor cells and may, therefore, prove useful in future regenerative therapies [26]. 

The junction between the ductal epithelium and adjacent acinar cells houses centroacinar/terminal ductal cells (CA/TD), which lack many markers of differentiated endocrine cells [27]. However, these cells are positive for progenitor cell transcripts including nestin and Sox9 [27]. Rovira and colleagues have recently shown that CA/TD cells are capable of spontaneously developing into endocrine or exocrine phenotypes and retain glucose-induced insulin secretion. Moreover, it was suggested that these cells contribute to the preservation of tissue homeostasis in the murine pancreas [27].

## 4. Directed Differentiation of Nonendocrine Progenitors

Given the common origin of the liver and pancreas and shared progenitor cells, the liver has been examined as an alternative source of islet progenitor cells. Under normal circumstances hepatocytes are able to regenerate and proliferate; however, chemical inhibition of this process results in the production of a liver progenitor cell population which is referred to as oval cells [28]. Oval cells share common features of pancreatic progenitor cells and may be directed towards an insulin-producing phenotype under appropriate culture conditions [29], including culture in high-glucose medium [30]. Hepatic expression of the Pdx-1 gene in the liver of streptozotocin-induced diabetic mice has been shown to result in the presence of insulin-positive cells in the liver [31, 32]. However, this approach has been limited firstly, by the toxicity associated with adenoviral delivery of the Pdx-1 gene [31] and, secondly, by the high level of mortality associated with Pdx-1 expression in the liver which lead to hepatic dysmorphogenesis [33] and autodigestion of hepatic cells which coexpressed exocrine enzymes and insulin [32]. 

In an attempt to overcome this complication, Kojima and colleagues used a transcription factor located downstream of Pdx-1 called B2/NeuroD to induce the neogenesis of islet cells expressing all four major islet hormones in the liver [32]. In a similar vein, the adenoviral delivery of Ngn3 in combination with a beta-cell growth factor called betacellulin to the liver of streptozotocin-induced diabetic mice resulted in the production of islet-like cells releasing insulin, glucagon, somatostatin, and pancreatic polypeptide [34]. In both studies, the resulting islet-like cells were reported to display glucose-stimulated insulin secretion and, following *in vivo* transplantation, reversed streptozotocin-induced diabetes for extended periods of time. Importantly, the beta-like cells derived following viral transfection of Ngn3 and betacellulin were found to originate from liver oval cells by lineage tracing [32, 34]. Very recently, it was found that transcription factors found in adult pancreatic cells, most notably NKX6.1, which has been shown to be essential in alpha- and beta-cell development in a variety of organisms [35–37], promotes Pdx-1-induced liver to beta-cell reprogramming, and such approaches may provide an alternative means of directing hepatic cells to a beta-cell phenotype [38].

## 5. Generation of Islet Cells from Embryonic Stem Cells (ESCs)

The recent FDA approval of the first clinical trial using human ESC in the United States has brought with it renewed confidence that stem cell-based therapies may become clinically tested. Given their pluripotent nature, ESCs were traditionally viewed as being a highly attractive source of material which could be differentially directed towards specific islet cell types by following established developmental pathways. However, the production of a single islet hormone-secreting or -expressing cell remains difficult. 

ESCs are isolated from the inner cell mass of a blastocyst. In this undifferentiated state, it has been reported that embryonic stem cells may express insulin naturally [39]. Cells containing the insulin gene may then be selected and directed towards a beta-cell phenotype through various culture conditions. Soria and colleagues were the first to clone insulin-secreting cells from undifferentiated ESCs via a “cell-trapping” technique [39]. This technique is based on the principle that a greater yield of differentiated cells will be obtained when a genetic marker is used. In this instance, ESCs are transfected with a chimeric construct which couples the insulin gene with a gene that confers drug resistance (i.e., neomycin). Therefore, cells which express the protein of interest (i.e., insulin) will also be positive for the neomycin resistance gene [39]. These cells displayed regulated insulin secretion in response to various secretagogues *in vitro*, while clusters of these clones were able to reverse hyperglycaemia induced by streptozotocin in mice. The cell-trapping technique was found to obtain a relatively pure population of insulin-positive cells expressing the beta-cell markers Pdx-1, Nkx6.1, insulin, glucokinase, glucose transporter 2 (GLUT-2), and Sur-1 [39]. However, the production of functional beta-cells capable of responding exclusively to glucose was not achieved. 

An alternative approach developed by Lumelsky and colleagues involves the production of insulin-secreting cells from ESCs derived from colonies of nestin-positive embryoid bodies [28]. Nestin is expressed in the embryo in developing neurons. Neural and islet cells share phenotypic similarities, and, therefore, nestin-positive cells are selected with the aim of differentiating towards an islet cell phenotype [40]. Following a novel five-stage differentiation process, the resulting insulin-producing cells were able to self-form clusters that were reported to be anatomically similar to rodent islets *in vivo *[28]. Further adaptations of this protocol showed that glucose-dependent insulinotropic peptide (GIP) treatment aided the differentiation process [41]. However, these cells also coexpressed other islet hormones, and concerns have been raised as to whether the altered culture conditions detailed in the protocol are sufficient to achieve a true beta-cell phenotype [42–45]. The detection of *de novo* insulin secretion from these differentiated stem cells has also proved difficult arising from the inherently high proportion of insulin added to the culture medium [45]. 

Subsequent studies have focused on altering culture conditions or differentiation process to produce insulin-secreting cells from rodent ESCs. Culturing mouse ESCs with inhibitors of phosphoinositide 3-kinase, an essential intracellular signaling regulator, yielded cells that resembled pancreatic beta-cells and which rescued glycaemic control in diabetes mice [46]. The regulated expression of Pdx-1 was also shown to aid *in vitro* differentiation of insulin-producing cells from ESCs [47]. Furthermore, it was shown that Pax4 overexpressing ESCs showed significant improvements in Ngn3, insulin, islet amyloid polypeptide, and Glut-2 mRNA levels [48]. Despite these improvements in culture models and differentiation processes, the production of a mature and glucose-responsive beta-cell from ESCs remains elusive. A summary of the culture conditions and differentiation processes used to generate insulin-secreting cells from ESC is given in Table 1.

The ESC studies described thus far have concentrated on the use of undifferentiated or partially differentiated ESCs [16–22]. Clinically, however, the use of partially differentiated cells is limited by the possibility of tumour formation. Rezania and colleagues sought to address this issue by developing a differentiation protocol utilizing feeder- and serum-free conditions to differentiate human ESCs into glucagon-secreting cells that resemble mature alpha-cells [49]. During early stages of the differentiation process, the cells were reported to coexpress glucagon and insulin. However, subsequent culture stages saw the reduction of beta-cell markers and fortification of alpha-cell characteristics including responsiveness to arginine [49]. Moreover, transplantation of these cells resulted in the release of fully processed and biologically active glucagon following fasting and amino acid stimulation [49]. It is hoped that this protocol may be further developed in order to produce mature beta-cells from ESCs.

## 6. Induced Pluripotent Stem Cells for Islet Cell Generation

**Figure 2:**
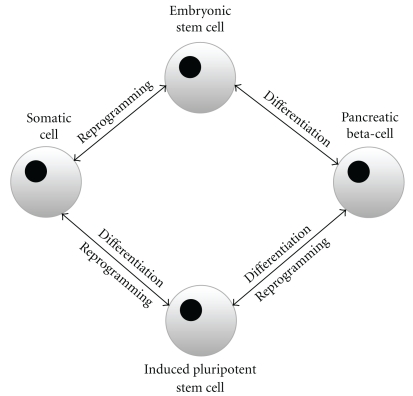
Directed differentiation of embryonic stem cells (ESCs) and induced pluripotent stem cells (iPSCs) towards a beta-cell phenotype. ESCs are derived from the inner cell mass of the blastocyst and have the potential to develop into any somatic cell lineage. Studies have shown that ESCs can be differentiated towards a beta-cell phenotype which releases insulin in response to stimuli including glucose. iPS cells are derived as a result of the directed reprogramming of somatic cells into a pluripotent phenotype. This generally involves the forced expression of a gene or transcription factor. iPSCs can be used to generate beta-like cells, while beta-cells themselves can be used to produce iPSCs for further expansion of the beta-cell population.

The directed reprogramming of somatic cells into a pluripotent phenotype is referred to as induced pluripotency and is outlined in Figure 2. In practice, this generally involves the forced expression of specific factors including, for example, transcription factors [50]. The resultant induced pluripotent stem cells (iPSCs) share common features with ESCs including high telomerase activity and hypomethylation of gene promoters [51, 52] but may be seen as preferable to ESCs as iPSCs can be patient specific, thereby, removing the likelihood of rejection. 

Recently the conversion of iPSCs originating from fibroblasts to pancreatic beta-like cells was achieved through a three-stage differentiation process [53]. Initially, embryoid bodies were formed from a single cell suspension of iPSC. Subsequently, the embryoid bodies were returned to adherent cultures and allowed to undergo further spontaneous differentiation [53]. The final stage involved directed differentiation in medium supplemented with laminin, insulin, and nicotinamide [53]. These cells shared many transcripts with native beta-cells including insulin, islet amyloid polypeptide, and Pdx-1. Furthermore, transplantation of these cells into diabetic mouse model provided long-term correction of hyperglycaemia with attainment of normal haemoglobin (Hb) A1c levels [53]. An additional study by Zhang and colleagues employed identical culture protocols to differentiate human ESCs and iPSCs into mature pancreatic insulin-producing cells [54]. iPSCs were induced from human fibroblasts into Pdx-1 expressing progenitor cells before final differentiation towards an insulin-secreting phenotype. The differentiated cells were positive for Pdx-1, MafA, GLUT2, insulin, and C-peptide [54]. These findings may open the possibility of using patient-specific iPSCs for cell replacement therapies in diabetes.

Interestingly, it has also been found that adult pancreatic beta-cells can be reprogrammed into iPSCs [55, 56]. Lentiviruses expressing the pluripotency markers Oct4, Sox2, c-myc, and Klf4 were introduced into tagged mature beta-cells, and their development was followed. It was found that transfected beta-cells produced iPSCs that were positive for markers of pluripotency and gave rise to a variety of cell types of all germ layers in chimeric animals [55]. This observation has been replicated using fibroblasts from patients with type 1 diabetes, which were induced to pluripotency and onwards to an insulin-secreting cell type [56]. 

While iPSCs offer great hope for stem cell-based therapies, the potential complications of this approach should not be overlooked. Recently, Lister et al. report that iPSCs display significant variations in reprogramming. Importantly, the differentiation of iPSCs to trophoblasts caused significant changes in DNA methylation that were maintained even after full differentiation has been completed [57].

## 7. Future Perspectives

Significant advancements have been made in the conversion of nonislet cells to islet hormone-secreting cells. The majority of research has focused on the conversion of non-beta-cells to beta-cells in an attempt to provide cellular transplants affording insulin secretion, and ideally glucose responsiveness, for the tight control of diabetes. Given the observations that homotypic cells interactions are sufficient to maintain normal patterns of insulin secretion [58–60], the production of pure beta-cell populations from ESC, progenitor cells, or iPS may well prove sufficient to restore glucose homeostasis. However, at present, an ESC-based approach to beta-cell generation is complicated by the heterogenous nature of the differentiated cells which express all four major islet hormones and lack of *de novo* insulin secretion [21, 22]. Furthermore, the unacceptable risk of teratoma formation [61] means that the therapeutic application of ESC-derived islet cells is unlikely at least in the near future. As such, further focus on feeder-free culture systems is required and may improve the likelihood of ESC-based therapies for the treatment of diabetes.

Initial studies on the use of endocrine and nonendocrine progenitor cells to produce insulin-secreting populations have proved promising. However, an incomplete understanding of the transcriptional regulation and debate over what constitutes a true islet progenitor as well as how beta-cells replicate and proliferate has made these findings difficult to interpret in terms of their suitability for therapeutic application. Recent work on iPSC has offered more hope for the development of a cell replacement therapy for diabetes. Moreover, there is a growing field of research on the directed differentiation of mesenchymal stem cells (MSCs) towards a beta-cell phenotype. While MSC populations are less expandable than ESC or iPS populations, they may be obtained from bone marrow or adipose tissue and, therefore, may be derived from the recipient and thus should alleviate the need for immunosuppression. A great deal of time and investment is currently being placed on new methods to track and monitor the viability of stem cells after transplantation. These include *in vivo *imaging techniques and cell labeling as reviewed in detail by Rodriguez-Porcel [62]. These advances will greatly aid with stem cell-based therapies for a host of different disorders. 

Despite the shortcomings associated with stem cell-based approaches for islet-cell generation, the need for a genuine cell replacement therapy for diabetes persists. The incidence of diabetes is rapidly increasing worldwide, warranting a range of approaches to tackle this life-threatening condition. The administration of exogenous insulin and incretin mimetics (see review by Irwin and Flatt [63]) can certainly alleviate hyperglycaemia in type 1 and poorly controlled type 2 diabetes. However, these therapeutic approaches do not offer an ideal long-term solution, and strict adherence to treatment regimes and regular glucose monitoring are required to prevent many of the devastating long-term complications of diabetes. Islet and whole pancreas transplantations are not a realistic large-scale solution. Therefore, the directed differentiation of nonislet cell types may offer the only large-scale alternative to produce a viable cure. While the current protocols require development, this research field is still in its infancy and is rapidly expanding with new developments appearing on almost a monthly basis. Moreover, the rapid advances in molecular and cellular biology provide the continual promise of exciting new mechanistic insights into cell development, differentiation, and survival. Accordingly, stem cell-based approaches offer great potential for the future and, with perseverance, may ultimately provide a cure for diabetes [64]. 

##  Review Criteria

The literature presented in this paper was compiled using PubMed and Ovid databases. No date or language restrictions were applied to the search criteria. Search terms used included embryonic stem cells, induced pluripotency, islet cells, insulin production, and diabetes mellitus.

## Figures and Tables

**Table 1:** Summary of approaches used to differentiate embryonic stem cells to insulin-producing cells.

	Origin	Differentiated from	Markers	Ref.
1	Mouse	Embryoid bodies (Hanging Drop Technique)	Pdx, Nkx6.1, insulin, GLUT2, glucokinase, SUR1	[39]
2	Mouse	Embryoid bodies	Nestin, Pdx1, Nkx6.1, Oct4, insulin, glucagon	[28]
3	Mouse	Embryoid bodies (addition of GIP to culture medium)	Nestin, Pdx1; Nkx6.1, Pct4, insulin, glucagon, GLUT2, SUR1, Kir6.2, GLP-1R	[41]
4	Human	Definitive endoderm	GSC, SOX17, FOX2A	[64]
5	Human	Embryoid bodies (use of knock-out serum replacement)	Maf6, Nkx6.1, Isl-1; NeuroD, Pdx-1, GLUT2, insulin, C-peptide	[53]
